# A PERMA model approach to well-being: a psychometric properties study

**DOI:** 10.1186/s40359-024-01909-0

**Published:** 2024-07-30

**Authors:** Maha Al-Hendawi, Ali Alodat, Suhail Al-Zoubi, Sefa Bulut

**Affiliations:** 1 Department of Psychological Sciences, College of Education, Qatar University, P.O.Box: 2713, Doha, Qatar; 2 Qatar University, Doha, Qatar; 3 Sultan Qaboos University, Muscat, Oman; 4 Ibn Haldun University, Istanbul, Turkey

**Keywords:** PERMA, Psychological health, Well-being, Adolescents, Behavior disorders

## Abstract

Psychological well-being influences all the facets of adolescent success. This study examined the psychometric properties of PERMA, a subjective well-being measure consisting of five domains: positive emotions, engagement, relationships, meaning, and accomplishment. The sample size consisted of 502 high school students from public schools in Qatar. Contrary to expectations, the five-factor structure for PERMA did not fit our data well; however, after refining the model by the errors of the covariances between items, the one-factor model was found to be applicable. Multivariate analysis revealed that socioeconomic status (SES) and level of academic performance were independently and positively associated with overall well-being scores, whereas internalizing and externalizing behaviors were negatively associated. Notably, no significant effects of gender or age on well-being were observed. These findings suggest that interventions specific to socioeconomic status, academic excellence, and internalizing and externalizing behaviors in the school setting can produce more effective outcomes concerning student well-being.

**Supplementary Information:**

The online version contains supplementary material available at 10.1186/s40359-024-01909-0.

## Introduction

Well-being is a concept derived from hedonic and eudaimonic perspectives [1, 2]. The former focuses on the fundamental needs of human beings and pain avoidance, whereas the latter focuses on the meaning and self-realization of their full potential [3–5]. Well-being encompasses not only the daily experience of positive emotions but also a state of effective functioning that enables an individual to realize their potential. It is a multifaceted concept that includes elements of emotional well-being, purposeful living, autonomy, fulfilling relationships, and overall life satisfaction. These components are integral to the physical, mental, and social aspects of life, contributing to a holistic sense of wellness [6].

Well-being has been found to have a significant relationship with positive mental health and overall success in life [1, 7], and many desired academic and social outcomes, such as increased educational and occupational success, and improved physical health, are associated with well-being [3, 5, 8]. To measure well-being, educators can look for the presence of resilience factors such as coping skills, perceived social support from peers and teachers, positive thinking patterns, and strong relationships.

As a measure of well-being, Seligman's PERMA model [8] has been found to be promising in several studies [3, 9–11]. It comprises five positive psychological elements, namely, positive emotion, engagement, relationships, meaning, and accomplishment, which lead to the acronym PERMA. Although the PERMA model is gaining wider acceptance, the existing literature provides inconsistent conclusions about the impact of sociodemographic, economic, and behavioral factors on well-being, as evaluated by the PERMA score. Given the variability in the available research, the factor structure and predictive and concurrent validity of the PERMA model of well-being require further investigation. Furthermore, literature on the impact of young people's mental, emotional, and behavioral disorders on their well-being is scant. Notably though, young people with mental, emotional, and behavioral disorders experience significant impacts on their well-being. Factors such as psychosocial challenges, poor health behaviors, and low life satisfaction contribute to their overall well-being [6, 12]. These individuals often face difficulties in coping with daily life, which can lead to elevated stress levels, anxious rumination, and dissatisfaction with information. It is crucial to address these issues and provide support to improve their well-being. Cheung and Dewa [13] drew attention to their struggles with daily life and the related stress and dissatisfaction. Addressing these challenges is critical [13]. Interventions that focus on enhancing coping skills, increasing parental emotional support, and promoting positive relationships can help mitigate the negative effects of mental, emotional, and behavioral disorders on young people's well-being [14].

Factors affecting well-being outcomes in Arab countries may differ from those in Western or Asian literature because of cultural, socio-political, and religious factors unique to the region [15]. Arab societies often emphasize strong familial ties, communities, and collective values, which can impact perceptions and experiences of well-being. Additionally, the varying sociopolitical landscapes and economic conditions in Arab countries can influence mental health and social attitudes towards well-being. Furthermore, religion plays a central role in Arab countries, affecting daily life and perspectives on mental health. However, to date, only one study of PERMA has been conducted in an Arab country. D'raven and Zaidi used secondary data from the United Arab Emirates (UAE) to examine the meaning of well-being and happiness in the UAE [16]; however, no study has been reported from Qatar that uses PERMA to gauge the well-being of adolescents. In particular, Qatar has undergone social, economic, and cultural transformation in the last two decades. Unavoidably, these advances have an influence on adolescents, who differ significantly from previous generations in several ways, including improved living standards and increased options to meet their needs. In addition, there is significant pressure to cope with peer pressure, social standards, and high standards of success. For example, secondary education students are under tremendous pressure to succeed [17]. Success is intricately related to life satisfaction and positive emotions; therefore, it is essential to understand the viewpoints and emotional states of students to build interventions that might improve their well-being [18]. To develop effective positive psychology programs and adolescent interventions, educators must first understand the factors that drive the desired positive outcomes in students [19].

Sociodemographic factors refer to the social and economic characteristics of a population [20]. These factors include, but are not limited to, income level, educational attainment, occupational status, family structure, race, ethnicity, and gender. Social and economic factors have a significant impact on children's behavioral problems. Typically, a lower socioeconomic status is associated with increased exposure to stressors, reduced access to resources, and a higher incidence of adverse health and developmental outcomes [21]. Academic performance is the extent to which a student, teacher, or institution achieves short- or long-term educational goals [22]. Cumulative GPA and the completion of educational benchmarks, such as secondary school diplomas and bachelor's degrees, represent academic performance. Academic performance is affected by a multitude of factors including, but not limited to, psychological well-being, social support systems, and one's socioeconomic background [23]. Internalizing behaviors are directed inward towards the self [24]. These behaviors are often psychologically based and can include anxiety, depression, withdrawal, and somatic complaints. Children who exhibit high levels of internalizing behaviors may struggle with emotional regulation and social interactions and may display a propensity to internalize distress as opposed to outwardly expressing distress. Externalizing behaviors are problem behaviors directed outward toward the external environment [25]. These can include aggressive, confrontational, or disruptive actions. Such behaviors are easily observable and can manifest as defiance, impulsivity, noncompliance, and other forms of antisocial conduct. These behavioral patterns can pose significant challenges to students’ social, academic, and behavioral development [26].

Children from families with a lower socioeconomic status (SES) tend to experience more mental health issues, which affects their social adaptation to school. According to Hosokawa et al. [27], there is a negative correlation between lower SES, measured by family income and parental education level, and behavioral problems in children [27]. This relationship suggests that children from disadvantaged backgrounds may face more challenges in regulating their behavior, leading to issues such as higher rates of internalizing and externalizing behavior. Interventions starting from early childhood education can lead to better academic and behavioral outcomes in schools [28].

With the understanding that students navigate increasingly complex social and cultural environments, it is vital that research fully captures their experiences. This study aimed to examine the well-being of high school students in Qatar and the effects of sociodemographic factors, academic performance, and internalizing and externalizing behaviors on the well-being of adolescents. The PERMA model, which includes elements of positive emotion, engagement, relationships, meaning, and achievement, is apt to meet this need. While research on adolescent well-being is scarce, particularly in the context of our study's locale, the all-encompassing framework of the PERMA model enables a thorough examination of this age group. Through this approach, our study is designed to uncover insights that could significantly improve adolescent well-being, facilitating their personal growth and fostering a smoother transition into productive adulthood.We investigated whether the five pillars of happiness in the PERMA model were reflected in what happiness meant to the participants.

## Materials and methods

### Participants, setting, and procedure

The study population consisted of high school students in Qatar who had attended government schools. This study used a cross-sectional design. In Qatar, government-funded secondary schools are typically segregated by gender, operating under the distinction of 'all-boys' or 'all-girls' institutions [29]. Four schools were strategically selected in conjunction with the Ministry of Education and Higher Education to ensure a representative sample that includes diverse socioeconomic backgrounds. The chosen schools, including two all-girls and two all-boys, were located in different regions in Doha, the nation's capital. Doha is the most densely populated area in Qatar, with over 80% of the country's population residing there, thus offering a broad demographic spectrum. The municipality of Al-Doha, where these schools are located, plays a crucial role in the educational landscape of the country due to its urban setting and accessibility to educational resources and facilities [29]. Students were invited to participate in the study at their respective schools through consent forms that were sent to their parents and students. The Ministry of Education and Higher Education sent text messages to students as reminder prompts in the Learning Management System to complete the questionnaire(s) and submit them via the link.

Two thousand ninety-five high school students were approached and 502 valid responses were received. Prior to data collection, we conducted a power analysis using G*Power 3.1 to determine the target sample size needed to detect effects in our planned analyses. For multiple linear regression with 7 predictor variables, an alpha of 0.05, and power of 0.80, the recommended sample size was 103 subjects. Our obtained sample of 502 students well exceeded this minimum threshold. The study was carried out according to the Declaration of Helsinki and was approved by the Institutional Review Board of Qatar University (approval code QU-IRB QU-IRB 1591-EA/21).

### Instruments

PERMA is a common measure of subjective well-being that consists of 15 items in five domains defined by Seligman [8]: positive emotion, engagement, relationships, meaning, and accomplishment. The following is a brief description of each of these five domains [30].

- Positive emotions (P): These are an important part of well-being and generally include tendencies toward feeling pleasure.
- Engagement (E): This refers to being attracted, interested, and involved in an activity or world.
- Relationships (R): This refers to feeling loved, supported, and valued by others.
- Meaning (M): This refers to a sense of purpose, the direction in which life is going, and the feeling that life is valuable.
- Accomplishment (A): This includes feelings of mastery and achievement, working towards and reaching goals, and a sense of being able to perform tasks.

The PERMA includes eight filler items that aim to control response biases. The eight component elements comprise one element that evaluates overall happiness; three elements of negative emotions that assess sadness, anger, and anxiety; one that assesses loneliness; and three that assess self-perceived physical health. In this study, only loneliness was used. It is important to note that loneliness, specifically, is a critical psychological factor with significant implications for mental health, particularly in adolescents [31, 32]. All items are scored on an 11-point Likert scale ranging from 0, which denotes "never," "terrible," and "not at all," to 10, which denotes "always," "excellent" and "completely," depending on the item. The three-item scores for each domain were averaged to produce a single domain score, ranging from 0 to 10 (with higher scores indicating greater well-being). The total PERMA score was calculated by averaging the scores for the 15 items. The higher the average, the more positive the function.

### Strengths and Difficulties Questionnaire (SDQ)

The SDQ was used to measure externalizing and internalizing behaviors of adolescents. This has been validated in the Arab population [29, 33–35]. Externalizing behaviors, which are outward-directed actions that include symptoms such as aggression or disruptive conduct, were quantified using a score ranging from 0 to 20. This score is the sum of the conduct and hyperactivity scales of the SDQ. On the other hand, internalizing behaviors, which are focused inward and often involve emotional distress or social withdrawal, were also scored from 0 to 20, based on the scales for emotional symptoms and peer problems of the SDQ.

### Externalizing differences

The externalizing score ranges from 0 to 20 and is the sum of the conduct and hyperactivity scales [36]. Participants were classified into the following four groups/levels based on their externalizing values.

- 0–8 is close to average
- 9–10 is slightly raised
- 11–12 is high
- 13 + is very high

### Internalizing differences

The internalizing score ranges from 0 to 20 and is the sum of the emotional and peer problems scales [36]. Participants were classified into the following four groups/levels based on their internalizing values:

- 0–6 is close to average
- 7–8 is slightly raised
- 9–10 is high
- 11 + is very high (covering approximately 5%)

### Procedure

#### Data collection

Schools were recruited to participate in the study, in collaboration with the Ministry of Education of Qatar. The researchers provided the collaborating schools with copies of the materials which included consent forms, the PERMA questionnaire, internalizing and externalizing SDQ items, and demographic information.

#### Translation of the PERMA Profiler

The Arabic version of the instrument was obtained from Abu-rayya and Sam [37], who used it to examine the Arab population in Australia. Further procedures were performed to ensure the appropriateness of the instrument, such as double-checking the English and Arabic translations and consulting faculty, teachers, and high school students on the questionnaires to ensure their accuracy and appropriateness. All agreed that they were appropriate for the context and population.

### Data analysis

The author obtained descriptive statistics from the participants, including age, gender, socioeconomic status, nationality, strands, and academic performance. The author also obtained descriptive data for each PERMA subscale or domain, consisting of three items, each of which was measured on a scale ranging from 0 to 10. Three item scores were averaged for each domain to produce a single domain score. Socioeconomic status (SES) was evaluated using a composite index that typically incorporates factors such as parental education, family income, and occupational status. Parental education was assessed by the highest level of schooling completed, family income was gauged through income brackets, occupational status was categorized based on a standard classification system, and pairwise deletion was chosen for handling missing values. The reliability of the scale was also tested. Cronbach’s alpha was determined to be a key indicator of internal consistency for each subscale and total well-being. The suitability of factor analysis was assessed using sample adequacy tests, including Bartlett's test of sphericity and the Kaiser–Meyer–Olkin (KMO) measure of sampling adequacy. Confirmatory factor analysis (CFA) was performed to verify the factorial validity of PERMA. The models were compared using chi-square (*χ*2), Comparative Fit Index (CFI), TLI, and mean squared error of approximation (RMSEA). Chi-square is sensitive to large sample sizes; therefore, model fit was considered acceptable if the CFI was > 0.95, the Root Mean Squared Error of Approximation (RMSEA) was < 0.06, and the Standardized Root Mean Square Residual (SRMR) was < 0.08 [5, 38]. Construct validity was assessed by examining Pearson's correlations between PERMA Profiler well-being scores and other theoretically relevant outcomes, where *r* = 0.00 -0.30 was considered a negligible correlation, *r* = 0.30 -0.50 was considered a weak association, *r* = 0.50 -0.70 was considered, a moderate correlation; and *r* > 0.70, a strong correlation. A multivariate analysis (MANOVA) was performed for gender, age, department, nationality, SES, academic performance, absence, externalizing behavior, and internalizing behavior as independent variables and five-domain PERMA as dependent variables. A multivariate regression analysis was performed to determine the factors influencing the overall PERMA score.

## Results

### Survey outcome and demographics

Participants represented three-grade high school levels:10th, 11th, and 12th. Males (64.1%) outnumbered females (35.9%) in the sample. The average age of the participants was 16.32 ± 1.12 years. Most students in the sample studied science, and more than 38% were Qatari. In terms of socioeconomic status, the majority of the participants (61.6%) were in the average group/level. Furthermore, according to the self-reported academic performance, students mostly had good or excellent academic performance.

### Reliability analysis

The internal reliability of each PERMA subscale was assessed using the Cronbach's alpha. Except for engagement, which had a reliability value of 0.57, all five subscales of the PERMA had acceptable reliability ratings of over 0.70. The Positive Emotion (P) subscale, which assesses the frequency and extent of positive emotions, includes items such as 'feeling joyful' (P1) and 'feeling contented' (P3), with a Cronbach's alpha of 0.91. Engagement (E) focused on immersion in activities, with items such as 'becoming absorbed in what you are doing' (E1), and had a lower alpha of 0.57. Relationships (R), measuring social support and satisfaction, included items such as 'feeling loved' (R2), showing good internal consistency with an alpha of 0.78. Meaning (M), exploring purpose in life, included 'leading a purposeful and meaningful life' (M1), with a high alpha of 0.90. Each subscale comprises three items, confirming its structure and internal consistency. The SDQ had a reliability value of 0.75.

### Factor analysis of the PERMA scale

Preliminary analyses revealed that the medians of the items were greater than seven for all items (Table 1). The absolute skewness (-1.65 to -1.01) and kurtosis (3.26–5.39) were within acceptable ranges [39]. These variables were subjected to principal axis factoring ( Table 2, Figure S1). The KMO analysis resulted in a significant sampling adequacy of 0.95, which was significant. Bartlett's test of sphericity (*χ*^2^ = 5919.544, *df* = 78,* p* < 0.001) was also significant.

**Table 1:** Mean, skewness, and kurtosis of the data

Code	Item	Mean	SD	Median	Skewness	Kurtosis
P1	Item 1: In general, how often do you feel joyful?	7.43	2.42	8	-1.12	3.92
P2	Item 2: In general, how often do you feel positive?	7.68	2.44	8	-1.27	4.08
P3	Item 3: In general, to what extent do you feel contented?	7.82	2.37	9	-1.24	3.94
E1	Item 4: How often do you become absorbed in what you are doing?	7.65	2.37	8	-1.24	4.15
E2	Item 5: In general: to what extent do you feel excited and interested in things?	7.55	2.42	8	-1.17	3.91
E3	Item 6: How often do you lose track of time while doing something you enjoy?	7.82	2.57	9	-1.36	4.23
R1	Item 7: To what extent do you receive help and support from others when you need	7.17	2.67	8	-1.01	3.26
R2	Item 8: To what extent do you feel loved?	8.12	2.34	9	-1.66	5.40
R3	Item 9: How satisfied are you with your personal relationships?	7.98	2.41	9	-1.56	5.00
M1	Item 10: In general, to what extent do you lead a purposeful and meaningful life	7.76	2.37	8	-1.33	4.40
M2	Item 11: In general, to what extent do you feel that what you do in your life is	7.85	2.31	8	-1.36	4.45
M3	Item 12: To what extent do you generally feel you have a sense of direction in life	7.60	2.34	8	-1.21	4.23
A1	Item 13: How much of the time do you feel you are making progress towards accomplishing your goals	7.60	2.33	8	-1.22	4.23
A2	Item 14: How often do you achieve the important goals you have set for yourself?	7.51	2.27	8	-1.09	3.88
A3	Item 15: How often are you able to handle your responsibilities?	7.57	2.39	8	-1.16	3.87

**Table 2:** Principal axis factoring analysis

Code	Factor 1	Factor 2	Uniqueness
P1	0.80	0.07	0.35
P2	0.84	0.03	0.30
P3	0.85	-0.01	0.29
E1	0.80	0.00	0.36
E2	0.77	0.04	0.40
E3	0.20	0.83	0.27
R1	0.57	0.52	0.41
R2	0.76	0.11	0.41
R3	0.82	-0.01	0.33
M1	0.85	-0.03	0.28
M2	0.85	-0.10	0.27
M3	0.83	-0.12	0.30
A1	0.84	-0.17	0.27
A2	0.81	-0.23	0.30
A3	0.72	-0.14	0.47

All factor loadings of the five PERMA subconstructs ranged from 0.20 to 0.85. The results showed that the factor loadings exceeded the desirable standard of 0.70, with the exception of E3 and R1. These items were removed to ensure an acceptable convergent validity. Moreover, correlations among the five PERMA sub-constructs ranged from 0.60 to 0.82, which exhibited acceptable discriminant validity. The correlation matrix for PERMA and the externalizing and internalizing variables are presented in Tables 3, S1, and S2, respectively.

**Table 3:** Correlation matrix for PERMA and externalizing and Internalizing variables

	**(1)**	**(2)**	**(3)**	**(4)**	**(5)**	**(6)**	**(7)**
(1) Emotion (P1,P2,P3)	-						
(2) Engagement (E1,E2,E3)	0.70*	-					
(3) Relationships (R1, R2,R3)	0.74*	0.64*	-				
(4) Meaning (M1,M2,M3)	0.75*	0.69*	0.72*	-			
(5) Accomplishment (A1,A2,A3)	0.70*	0.61*	0.65*	0.82*	-		
(6) Internalizing Behavior Score	-0.46*	-0.30*	-0.36*	-0.43*	-0.49*	-	
(7) Internalizing Behavior Score	-0.57*	-0.43*	-0.51*	-0.54*	-0.51*	0.61*	-

^*^p < *0.1*

**Fig. 1:**
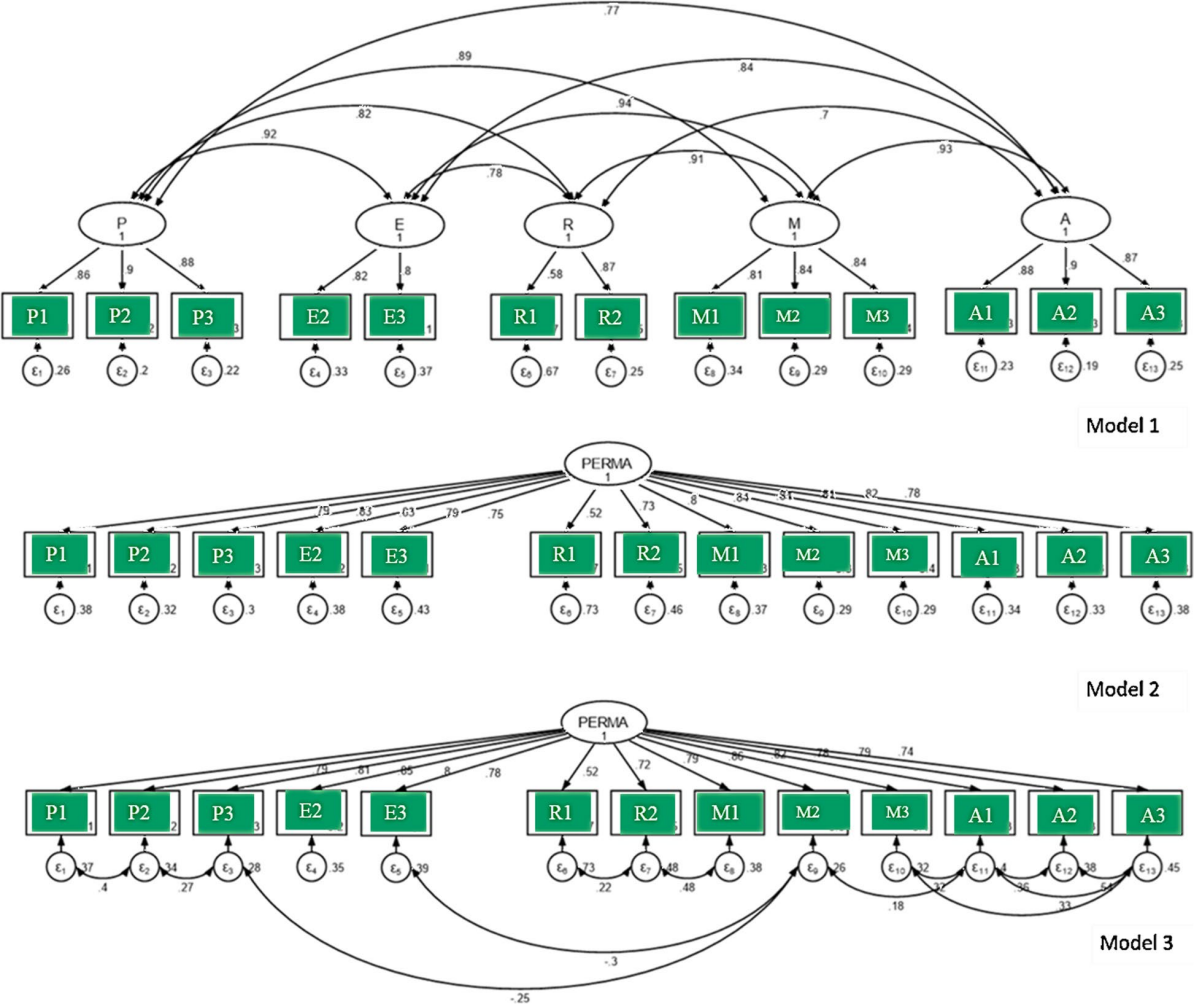
Different factor models along with coefficients

First, we defined an inter-correlated five-factor model (Fig. 1). Second, we tested the theoretically competing models and compared them with the five-factor model according to goodness-of-fit indices. Second-order latent variables can be used as explanatory variables for endogenous variables such as positive emotion (P), engagement (E), relationship (R), meaning (M), and accomplishment (A). Figure 1 illustrates the results of the hypothesized second-order factorial structure for PERMA. The five-factor model did not fit the data well (*χ*^2^ (90) = 321.0, *p* < 0.001, *CFI* = 0.95, *TLI* = 0.94, *RMSEA* = 0.095, *SRMR* = 0.03). Then, we explored a single-factor model. It also did not fit the data well (*χ2* (90) = 829.0, *p* < 0.001, *TLI* = 0.85, CFI = 0.87, *RMSEA* = 0.15, *SRMR* = 0.05). However, a single-factor modified model with covariances was found to fit PERMA (χ2 (90) = 160.0, *p* < 0.001, *TLI* = 0.97, *CFI* = 0.98, *RMSEA* = 0.06, *SRMR* = 0.03). Our modifications included specifying particular covariances between item pairs in the model to reflect the significant correlations observed in our actual data. For example, we found a strong positive covariance between items P1 ('feeling joyful') and P2 ('feeling positive') at 0.4, and a moderate positive covariance between M2 ('feeling what one does is valuable') and A2 ('achieving personal goals') at 0.33. Additionally, items P2 ('feeling positive') and P3 ('feeling contented') showed a moderate positive covariance of 0.27. Interestingly, negative covariances emerged between P3 ('feeling contented') and M1 ('leading a purposeful life') at -0.25, and between E2 ('feeling excited and interested') and M1 at -0.3. Incorporating these specific covariances significantly improved the model fit over a more restrictive single-factor model without cross loadings. Therefore, the CFA model presented in Model 3 was the final measurement model indicating the PERMA structure in the present population.

### Parameters affecting PERMA

#### Gender

The medians of the five domains were calculated for each gender (Table 4). The averages for PERMA and its components, boys and girls, were very similar, implying small and non-significant differences (*p* > 0.05). The Externalizing Behavior Score did not differ between boys and girls; however, internalizing behavior scores were higher in girls (*p* = 0.005). We also calculated Cohen's d for internalizing behavior scores, which revealed an effect size of -0.259. This value indicates a small but noticeable difference in internalizing behaviors between genders, with girls scoring slightly higher than boys did. Approximately 50% of the students came from the science department, followed by the arts and technology department. There were no statistically significant differences in the distribution of females and males in these departments (*p* = 0.47) or in financial conditions (*p* = 0.14). The academic performance pattern differed between boys and girls, with more girls having an excellent level of academic performance (boys: 24% vs. girls: 51.3%, *p* < 0.01). Similarly, females were less frequently absent from school (*p* = 0.04).

**Table 4:** Patient demographic, PERMA, and SDQ characteristics stratified by gender

	Boys	Girls	
Parameter	(*N* = 338)	(*N* = 195)	*p*-value
PERMA (Average)			.87
Median (Q1, Q3)	112.7 (97.4, 127.6)	113.6 (94.4, 127.6)	
Emotion (Average)			.59
Median (Q1, Q3)	8.0 (6.7, 9.3)	8.3 (6.3, 9.3)	
Engagement(Average)			.94
Median (Q1, Q3)	8.0 (6.7, 9.0)	8.0 (6.7, 9.0)	
Relationships Average			.36
Median (Q1, Q3)	8.3 (7.0, 9.3)	8.3 (6.7, 9.3)	
Meaning Average			.53
Median (Q1, Q3)	8.3 (6.7, 9.3)	8.3 (7.0, 9.3)	
Accomplishments Average			.35
Median (Q1, Q3)	8.0 (6.3, 9.0)	8.0 (6.3, 9.0)	
Externalizing Behavior Score			.30
Median (Q1, Q3)	4.0 (3.0, 7.0)	5.0 (3.0, 7.0)	
Internalizing Behavior Score			.005
Median (Q1, Q3)	5.0 (3.0, 7.0)	6.0 (3.0, 9.0)	
Department			.47
Science	175 (51.8%)	106 (54.4%)	
Arts	122 (36.1%)	72 (36.9%)	
Technology	41 (12.1%)	17 (8.7%)	
Nationality			.19
Qatari	137 (40.5%)	68 (34.9%)	
Non-Qatari	201 (59.5%)	127 (65.1%)	
Do you consider the economic/financial condition of your family?			.14
Below Average	64 (18.9%)	41 (21.0%)	
Average	202 (59.8%)	126 (64.6%)	
Above Average	72 (21.3%)	28 (14.4%)	
What is your academic level?			< .001
Below Average	19 (5.6%)	2 (1.0%)	
Average	65 (19.2%)	16 (8.2%)	
Good	139 (41.1%)	77 (39.5%)	
Excellent	115 (34.0%)	100 (51.3%)	
Are you absent from school?			.04
Often	8 (2.4%)	4 (2.1%)	
Sometimes	66 (19.5%)	20 (10.3%)	
Rarely	113 (33.4%)	72 (36.9%)	
Never	151 (44.7%)	99 (50.8%)	

*PERMA* Positive Emotion, Engagement, Relationships, Meaning, and Accomplishment

#### Age

Age groups were stratified into middle ( 14–16 years) and senior ( 17–19 years) age groups. There was no difference in the overall PERMA score, although engagement was slightly lower in the senior group (*p* = 0.02) (Table S2). There were no significant differences in academic performance, nationality, or department (all *p* > 0.05).

#### Socioeconomic difference

**Fig. 2:**
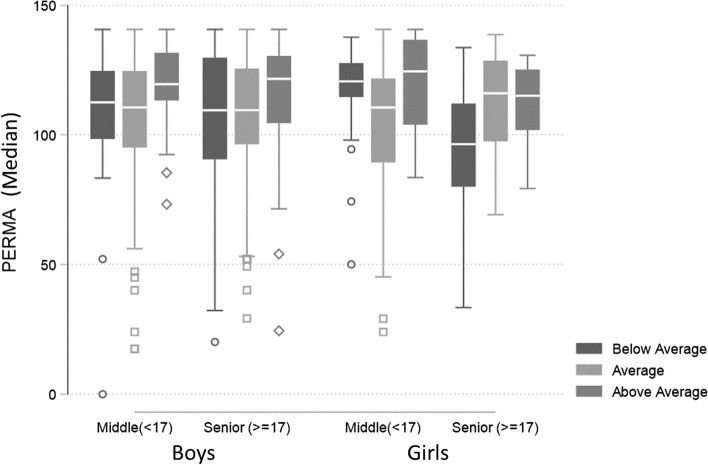
Median PERMA score in different subgroups

The results presented in Fig. 2 and Table 3 show some differences between the socioeconomic groups. The values of PERMA and its components in the "Above Average" group were higher than those in the other two socioeconomic groups. The Externalizing Behavior Score did not vary among the groups, although the Internalizing Behavior Score was lower in the above-average group.

#### Academic differences

The results presented in Table S4 show some differences between academic performance groups at the univariate level. The values of the "Above Average" group are higher than those of the other three groups, especially the "Below Average" group. For example, the mean of the "Excellent" group in the accomplishment domain is 8.17, while it is 4.30 for the "Below Average" group.

#### MANOVA

The author performed a MANOVA using gender, age, nationality, SES, academic performance, absenteeism, externalizing subscale, and internalizing subscale as independent variables, and the five domains of PERMA as dependent variables (Table 5). Wilks' lambda was nonsignificant for gender, age, nationality, and absenteeism (all *p* > 0.05). The findings of the multivariate analysis of SES, academic performance, externalization, and internalization, which were statistically significant (*p* < 0.05), corroborated the results of the univariate analysis.

**Table 5:** MANOVA results of the five PERMA domains over different SDQ and demographic characteristics

Variable	Statistics		df	F(df1,df2) = F			Prob > F
Gender	W	.99	1	5	481	.49	.78
	P	.01		5	481	.49	.78
	L	.01		5	481	.49	.78
	R	.01		5	481	.49	.78
Age	W	.98	1	5	481	1.5	.18
	P	.01		5	481	1.5	.18
	L	.01		5	481	1.5	.18
	R	.01		5	481	1.5	.18
Department	W	.96	2	10	962	1.85	.04
	P	.03		10	964	1.85	.04
	L	.03		10	960	1.86	.04
	R	.03		5	482	3.27	.01
Nationality	W	.99	1	5	481	.46	.80
	P	.01		5	481	.46	.81
	L	.01		5	481	.46	.80
	R	.01		5	481	.46	.80
SES	W	.96	2	10	962	1.91	.04
	P	.04		10	964	1.91	.04
	L	.04		10	960	1.91	.04
	R	.03		5	482	2.72	.01
Academic level	W	.91	3	15	1328.2	3.05	< .001
	P	.09		15	1449	3.01	< .001
	L	.09		15	1439	3.08	< .001
	R	.07		5	483	7.23	< .001
Absent	W	.95	3	15	1328.2	1.37	.15
	P	.04		15	1449	1.37	.15
	L	.04		15	1439	1.37	.15
	R	.02		5	483	2.18	.05
Externalizing Behavior Score	W	.75	16	80	2319.8	1.74	< .001
	P	.26		80	2425	1.71	< .001
	L	.29		80	2397	1.76	< .001
	R	.14		16	485	4.43	< .001
Internalizing Behavior Score	W	.60	18	90	2338	2.8	< .001
	P	.44		90	2425	2.65	< .001
	L	.55		90	2397	2.95	< .001
	R	.35		18	483	9.45	< .001

*L* Lawley-Hotelling Trace, *P* Pillai's Trace, *R* Roy's Largest Root, *SES* Socioeconomic Status, *W* Wilks' Lambda

#### Multivariate regression

To assess the effects of SES, academic performance, absenteeism, age, gender, externalization, and internalization subscales on PERMA, a multivariate regression was performed using PERMA as a dependent variable (Table 6). The results revealed that SES (4.0 [1.2–6,8, *p* < 0.001]) and academic performance (3.4[1.2–5.7], *p* < 0.001) were associated with a higher PERMA score, while the externalization subscale (-0.995[-1.6 to -0.8], *p* < 0.001) and internalizing subscale (-3.5[-4.1 to -2.8], *p* < 0.001) had a profound negative correlation with the PERMA scale.

**Table 6:** Regression Analysis Results for Predicting PERMA Scores

Variable	Coefficient (95% CI)	*p*-value
**Socioeconomic Status (SES) [Reference: Below Average]**		
Average	1.258945 [-3.258429 to 5.776319]	.584
Above Average	7.774642 [1.852203 to 13.69708]	.010
**Academic Level [Reference: Below Average]**		
Average	10.65735 [0.5089734 to 20.80574]	.040
Good	12.24121 [2.546807 to 21.93562]	.013
Excellent	15.70734 [5.734301 to 25.68039]	.002
**Department [Reference: Science]**		
Arts	0.265198 [-3.999521 to 4.529917]	.903
Technology	-3.442567 [-9.340364 to 2.455231]	.252
**Nationality [Reference: Qatari]**		
Non-Qatari	-1.647539 [-5.868835 to 2.573757]	.444
**Absent [Reference: Often]**		
Sometimes	-0.9135597 [-13.61436 to 11.78724]	.888
Rarely	-0.383415 [-12.61257 to 11.84574]	.951
Never	0.7810686 [-11.58513 to 13.14727]	.901
**Student Age**	-1.23508 [-2.840329 to 0.3701683]	.131
**Gender [Reference: Male]**	2.496442 [-1.261769 to 6.254653]	.192
**Externalizing Subscale Total**	-1.023114 [-1.713475 to -0.3327529]	.004
**Internalizing Subscale Total**	-3.397218 [-4.045553 to -2.748884]	< .001

*CI* Confidence Interval, *PERMA* Positive Emotion, Engagement, Relationships, Meaning, and Accomplishment, *SES* Socioeconomic Status, Reference categories are indicated in brackets next to each variable category

## Discussion

This study examined the reliability and validity of PERMA as a measure of adolescent well-being in Qatar and assessed the factors that might influence PERMA scores. Our results support the convergent and divergent validity of the scale. Internal consistency was good for the scale and its subscales, except for the engagement subscale. The Engagement scale's lower reliability could stem from the diverse nature of the items, possibly reflecting a broad spectrum of engagement aspects. Our study did not find any effect of gender or age on PERMA scores, although SES and academic performance significantly affected PERMA scores. Contrary to expectations, a five- or one-factor structure did not fit the data well. However, after refining the model by the errors of the covariances between items, the one-factor model was found to fit well, pointing towards the overlap of different items in the P, E, R, M, and A subscales.

It is essential to highlight that a five-factor model was proposed in the development and early validation of PERMA. However, in several other studies, the data did not fit a five-factor model. For example, only four of the five PERMA components were applicable to a study of Australian students between the ages of 13 and 18 [40]. In another validation study, Cobo-Rendón et al. [41] found a good adjustment of the PERMA model in a sample of Chilean students, although Cronbach's alpha of the engagement dimension was low. Watanabe et al. verified that both the unifactorial and five-factor models showed poor adjustment in a sample of Japanese workers, although the latter had the best fit. In the German population, single-factor, higher-order, five-factor, and two-factor models have been examined, and the five-factor model showed the best adjustment [11]. The differences in the validation of the PERMA model in different studies may be because people's views on well-being are increasingly diverse and can change with age or interactions with others [42]. In particular, Seligman argued that it would be incorrect to assume that PERMA constitutes a different kind of well-being than just its building blocks [43].

Sometimes, the characteristics of the sample may also cause unexpected results, such as data that do not conform to the predicted structure. The individuals in our sample reported considerably high PERMA well-being and subscale scores and significant variances in PERMA depending on SES and academic level. These variations distinguish our group from typical populations in several ways, and are explained by Qatar's unique demographics. Wammerl et al. [11] argued that the rejection of the single-factor model supports the multidimensional nature of PERMA [11]. In contrast, Rya et al., who investigated the psychometric qualities of PERMA in Australian adults [5], found that their data, like ours, did not conform to either a one- or five-factor framework. These authors conducted a bivariate item correlation study and demonstrated that the Accomplishment and Engagement subscales were likely causes of discordance since their item correlations were less than *r* = 0.50. None of the item correlations in our study were less than 0.5. For the correlation between achievement and meaning, r was 0.82, and item correlations were above 0.7 for meaning-emotions, meaning-relationship, and relationship-emotions. Notably, the factor loadings of E3 and R1 were low and these items were removed from the models.

Ray et al. mentioned that E1 and E3 refer to a single affective state and further argued that E2 might capture respondents' transient levels of excitement rather than a stable level of occupation with or attention to a task, which is how the construct is operationalized. In our study, the correlation between R1-R2 and R1-R3 was low, whereas R2-R3 was good. The observed correlations among items R2 ('To what extent do you feel loved?') R3 ('How satisfied are you with your personal relationships?'), and R1 ('To what extent do you receive help and support from others when you need?”) in the PERMA model might be influenced by a broader range of factors than initially considered. Specifically, R2 and R3, while primarily assessing the quality of interpersonal relationships, may also encapsulate feelings of being loved or connected in a wider sense, including spiritual connections, emotional bonds with pets, and self-love. Similarly, R1's focus on receiving help and support could extend beyond personal relationships to include community support systems or institutional assistance.'

The present study also examined the relationship between socioeconomic factors and the PERMA scores. Our results indicate that SES is independently associated with PERMA scores. According to Shoshani et al. [44], adolescents' well-being is affected by their socioeconomic status, with students living in poverty experiencing fewer positive emotions, more negative emotions, and fewer peer relationships [44]. Furthermore, in D'raven and Zaidi's [16] investigation of the preconditions of happiness in the UAE, when participants were asked what makes them happy, the phrase "economic conditions" was mentioned [16]. The authors assumed that socioeconomic factors influenced each of the five PERMA domains.

For example, in the domain of positive emotions, the students were asked about their feelings of joy. Having a higher-than-average income usually allows people to spend money on things that make them happy (i.e., items or experiences). Research indicates that beyond a certain income level, further financial gains do not reliably improve general happiness or life satisfaction [45]. This suggests a complex relationship between income and subjective well-being, where financial security facilitates happiness to an extent, but a higher income does not necessarily align with greater happiness. Quantitative self-reported joy may correlate more directly with disposable income, but broader well-being depends on many additional social, psychological, and lifestyle factors beyond purchasing power alone [46]. In terms of relationships, students were asked if they received help when needed. The findings revealed that having a higher socioeconomic status improves access to necessary assistance (e.g., private tutors). Questions regarding a sense of direction were asked to the students and if they could achieve their goals and handle their responsibilities in the domains of meaning and achievement, respectively. Naturally, higher socioeconomic status confers privileges and facilitates an individual's path toward their desired direction and achievement of their goals. Another study in India found that children in private schools had significantly better and more positive mental health scores than those in government schools; however, depression was more prevalent among urban adolescents than among rural adolescents [47]. One potential explanation is that students in private schools may experience reduced financial stress, better infrastructure, and a more attentive educational environment. This could enable greater psychological well-being. Meanwhile, higher rates of depression among urban youth compared to rural populations could be linked to increased competition and peer pressure, more exposure to socioeconomic disparities, family dynamics shifting towards nuclear households, or relative comparisons to higher local standards of living. Similarly, Shoshani et al. [44] demonstrated the influence of socioeconomic status on student well-being, where adolescents below the poverty line showed lower levels of positive emotions and peer relations [44]. They also found that students below the poverty line had reduced levels of cognitive engagement at school, and were rated by teachers as having reduced emotional engagement.

Our findings also indicated a correlation between academic performance and PERMA scores. The group with an "Excellent" academic performance scored significantly higher than the other groups. Schools and grades are important in high school students' lives as they can determine their future. Further studies are needed in the fields of well-being and PERMA applications. Tansey et al. [19] examined academic satisfaction among college students with disabilities, and the PERMA model revealed a positive association with factors associated with college success and a negative association with difficulties such as stress and relationship problems [19]. Shoshani et al. [44] demonstrated an association between grade level and student scores, where older students tended to score low in "behavioral and cognitive engagement in school, " in addition to having lower scores [44].

Generally, well-being scores are strongly associated with SES and academic performance. However, contrary to expectations, no associations were found between PERMA scores and age or gender. Several factors might explain this unexpected outcome. The decline in facets of well-being, including PERMA elements (positive emotions, engagement, relationships, meaning, and achievement), as noted in some studies, typically refers to trends observed across the entire lifespan, not just during adolescence. This decline is often attributed to various life challenges and transitions that occur as individuals age, such as health issues, changes in social roles, and environmental changes. However, it is important to note that this trend can vary widely among individuals and is influenced by many factors, including cultural, socioeconomic, and personal circumstances [10, 44]. Such effects could be due to individuals' desire to please others and family members in collectivist societies, which place even more stress and pressure on high school students as they age [16].

Additionally, statistically significant differences between the well-being of the middle and junior groups and the junior and senior groups were reported, indicating that younger students had better mental health than the middle and senior groups [10]. This could be attributed to the increased pressure that students face in higher classes, as their school performance is regarded as a high stake. In our study, although overall PERMA was slightly lower in the senior group, the difference was not statistically significant. Of the different subscales, only engagement was significantly lower in the senior group; the MANOVA and multiple regression did not reveal any effect of age on the overall levels of PERMA or its subscales. Furthermore, there were no differences in externalizing and internalizing behavior scores between the senior and middle-aged groups. In particular, previous research has produced mixed results, with some indicating that older students report lower levels of well-being and greater levels of ill being, suggesting that age differences may not influence all elements of well-being [10, 48, 49].

In our case, no effect of gender was observed on the PERMA scale or any subscale. Multiple studies have shown that boys perform better than girls in PERMA, and the gender intensification theory hypothesizes that some of these differences may be caused by the high prevalence of negative body image among adolescent females [10, 44]. First, structural factors refer to the different opportunities and arrangements provided to boys and girls; second, socioeconomic factors refer to the different expectations placed on boys and girls; and third, biological differences create different capabilities in males and females [50, 51]. Shoshani et al. admitted that they were unsure whether males and females experienced different levels of negative emotions or whether this difference was simply due to males' willingness to suppress and keep their negative emotions and distress hidden as opposed to females, who are more expressive of their emotions [44]. In the absence of a gender effect on PERMA in our study, a possible explanation could be that since females made up only about one-third of the total sample in this study, the imbalance in gender distribution minimized gender differences. Previous studies have demonstrated that gender differences are inconsistent across all aspects of well-being. Cultural and religious variables in Qatar may have been responsible for these outcomes. Further research is required to confirm these findings. Given the many components of well-being, the environmental and individual factors that can influence well-being, and the limitations of each study, identifying the causes of this disparity is challenging. Interestingly, Burke and Minton [10] discovered that while gender differences are evident in late adolescence, they diminish in early adulthood [10]. Late adolescence typically refers to the age range of 15–19 years, while early adulthood generally encompasses the 20–29 year age group. This diminishing gender gap in well-being may partly stem from issues such as body image concerns, which are more prevalent among adolescent girls, but tend to decrease as they transition into adulthood. This age-specific observation underscores the dynamic nature of well-being across different life stages, which is influenced by a range of developmental and sociocultural factors.

## Conclusions

Measuring population-level well-being is a leading research priority. In our study, which is the first from Qatar, consistent correlations in the expected direction with convergent and divergent conceptions indicate the potential for the practical application of PERMA. However, our results challenge the five-factor structure of the PERMA Profiler and favor a single-factor model with covariances among items. Our findings indicate that SES and academic success favorably affect well-being. In the context of the Qatari educational system, these findings advocate particular actions to promote the well-being of students of lower SES and those with lower academic excellence. Further studies are needed to determine indicators of other possible developmental, psychological, and systemic aspects that affect student well-being in Qatar.

### Strengths & limitations

The strengths of this study include the fact that it is the first to use the PERMA Profiler to assess the well-being of adolescents in Qatar and elucidate the associations between socioeconomic standing, educational achievement, and PERMA. This study has a few limitations. In this study, we used data from high school students from Qatar enrolled in public institutions as our sample. However, caution should be exercised when extrapolating these findings to other student groups. It is vital to replicate these studies by using different populations and contexts. For example, studies with geographic focus can provide information on regional variations and commonalities. The low response rate also limits the generalizability of our findings. It may be noted that the low response rate in our study can be attributed to many participants' unavailability or unwillingness to participate as well as factors such as a lack of incentives for participants and a cultural reluctance to share information about one's own and one's family's circumstances, despite assurances of confidentiality. It should also be noted that the practice of responding to research is still nascent in the country, and it is challenging to obtain support to gather and publish data on public or private education, or other sectors of Arab societies. Thus, it is common for individuals to avoid participating in research studies. In this study, the response rate was approximately 24%, which is quite low but not unusual for survey research conducted in school settings. However, the potential for nonresponse bias exists if there are systematic differences between students who choose to participate and those who do not. We have added this caveat regarding generalizability to the limitations. Another limitation of our study was that the ratio of boys to girls was disproportionate, with boys comprising nearly two-thirds of the sample. For a better comparison of the results on gender differences, the sample should preferably have an equal gender distribution. It should be noted that the engagement subscale had the lowest internal consistency among all the PERMA subscales. Limited internal consistency may be a sign of problematic item wording or item sampling; for instance, it could mean that respondents misinterpreted the items and gave varying responses to the same scale of items or that the questions did not adequately sample the construct of engagement. Researchers should be cautious when interpreting engagement ratings in the interim. Due to the lack of literature on the subject in the Arab region, it is not possible to obtain data on the well-being of Qatar's neighbors to identify patterns or irregularities as well as the reasons for them. This would have helped all interested parties in the Arab region to collaborate and develop appropriate plans to create supportive environments for adolescents and promote their well-being. As this study utilized cross-sectional survey data, causal inferences cannot be made regarding the relationships observed between PERMA dimensions, academic performance, and internalizing/externalizing behaviors. Specifically, we cannot ascertain the temporality or directionality of the effects between the variables. Thus, a limitation of the current analysis is the inability to establish causal links between factors, such as positive emotions, engagement, academic achievement, and behavioral outcomes, without multiple time points. Future longitudinal tracking is valuable for delineating potential causal pathways.

### Future studies

Future studies should consider longitudinal designs to track the evolution of well-being over time. This approach provides valuable insights into how well-being changes and develops, especially in response to life events and transitions. Investigating the efficacy of the PERMA model across various cultural and socioeconomic groups will further enhance our understanding of its applicability and relevance in diverse settings. In addition, there is a need to focus on the engagement subscale of the PERMA model. Future research should aim to refine measurement techniques, such as improving translation methods and rephrasing questions, to ensure greater accuracy and consistency. Moreover, as this study utilized cross-sectional survey data, causal inferences could not be made regarding the relationships observed between PERMA dimensions, academic performance, and internalizing/externalizing behaviors. Specifically, we cannot ascertain the temporality or directionality of the effects between the variables. Thus, a limitation of the current analysis is the inability to establish causal links between factors, such as positive emotions, engagement, academic achievement, and behavioral outcomes, without multiple time points. Future longitudinal tracking is valuable for delineating potential causal pathways. Exploring these areas will significantly contribute to the field of well-being research by offering a more comprehensive understanding of the factors that influence well-being across different populations and contexts.

### Practical implications

This study provides important insights into the well-being of high school students in Qatar, who attend government schools. Our findings can play an essential role in monitoring well-being status and developing policies that enhance students' well-being levels by designing specific well-being programs. For example, our study identified SES as being independent of overall PERMA scores. Educational policy may contribute to such effects and provide specific interventions to improve the well-being of economically less-privileged students. Additionally, students who cannot achieve academic excellence may benefit from specific measures such as moral support and motivation, and stress the importance of holistic education. Our study indicates that turning to Western findings or international methods to achieve well-being in Qatar is insufficient because many of their findings may be irrelevant or inapplicable to Arab society. For example, Qatar is highly diverse in terms of both cultural identity and country of birth.. If students perform poorly in social relationships, the school could organize a cultural day to encourage them to introduce their cultural background, break the ice between students, and promote their participation. Therefore, adapting the PERMA model to local needs and requirements may allow adolescents to meet their well-being needs. The well-being of young people must be developed, focusing on talent, strengths, potential, and positive attitudes to ensure the continuous affluence of Qatar's capacity.

### Supplementary Information

Supplementary  Material 1. 

## Data Availability

Data are not publicly available due to ethical restrictions and contain information that could compromise the privacy of the research participants.
