# Supplementary material for: A PERMA model approach to well-being: a psychometric properties study
Source: BMC Psychol. 2024 Jul 30;12:414. doi: 10.1186/s40359-024-01909-0 (PMC11290191; doi:10.1186/s40359-024-01909-0)
Supplement: Supplementary file 1 — Supplementary Material 1. [file 40359_2024_1909_MOESM1_ESM.docx]

Supplementary figures

Figure S1: Scree plot of PCA

## Figure S2: Correlation plots between different PERMA and SDQ parameters

Supplementary tables

## Table S1: Pairwise correlations between PERMA items

| Variables | (1) | (2) | (3) | (4) | (5) | (6) | (7) | (8) | (9) | (10) | (11) | (12) | (13) | (14) |
| --- | --- | --- | --- | --- | --- | --- | --- | --- | --- | --- | --- | --- | --- | --- |
| (1) x1 | 1.000 |  |  |  |  |  |  |  |  |  |  |  |  |  |
| (2) x2 | 0.795* | 1.000 |  |  |  |  |  |  |  |  |  |  |  |  |
| (3) x3 | 0.724* | 0.791* | 1.000 |  |  |  |  |  |  |  |  |  |  |  |
| (4) x4 | 0.624* | 0.621* | 0.709* | 1.000 |  |  |  |  |  |  |  |  |  |  |
| (5) x5 | 0.666* | 0.657* | 0.659* | 0.650* | 1.000 |  |  |  |  |  |  |  |  |  |
| (6) x7 | 0.453* | 0.452* | 0.427* | 0.426* | 0.391* | 1.000 |  |  |  |  |  |  |  |  |
| (7) x8 | 0.618* | 0.609* | 0.635* | 0.548* | 0.510* | 0.500* | 1.000 |  |  |  |  |  |  |  |
| (8) x9 | 0.663* | 0.653* | 0.668* | 0.595* | 0.601* | 0.404* | 0.772* | 1.000 |  |  |  |  |  |  |
| (9) x10 | 0.637* | 0.681* | 0.662* | 0.676* | 0.583* | 0.469* | 0.635* | 0.692* | 1.000 |  |  |  |  |  |
| (10) x11 | 0.604* | 0.640* | 0.656* | 0.671* | 0.643* | 0.423* | 0.587* | 0.659* | 0.717* | 1.000 |  |  |  |  |
| (11) x12 | 0.555* | 0.601* | 0.609* | 0.627* | 0.605* | 0.402* | 0.522* | 0.601* | 0.729* | 0.779* | 1.000 |  |  |  |
| (12) x13 | 0.569* | 0.629* | 0.656* | 0.637* | 0.579* | 0.379* | 0.529* | 0.606* | 0.703* | 0.724* | 0.771* | 1.000 |  |  |
| (13) x14 | 0.542* | 0.600* | 0.603* | 0.590* | 0.539* | 0.348* | 0.529* | 0.581* | 0.662* | 0.708* | 0.741* | 0.809* | 1.000 |  |
| (14) x15 | 0.463* | 0.542* | 0.560* | 0.500* | 0.470* | 0.369* | 0.520* | 0.555* | 0.561* | 0.586* | 0.581* | 0.640* | 0.672* | 1.000 |
| **** p<0.01, ** p<0.05, * p<0.1* | | | | | | | | | | | | | | |

## Table S2: Patient demographic, PERMA and SDQ characteristics stratified by age

| \|  \| Middle(<17) \| Senior (>=17) \|  \| \| --- \| --- \| --- \| --- \| \|  \| (N = 319) \| (N = 214) \| p-value \| |
| --- | --- | --- | --- | --- | --- | --- | --- | --- |
| \| PERMA (Average) \|  \|  \| 0.595 \| \| --- \| --- \| --- \| --- \| \| Median (Q1, Q3) \| 113.3 (97.4, 127.5) \| 112.5 (94.4, 127.7) \|  \| \| Emotion(Average) \|  \|  \| 0.630 \| \| Median (Q1, Q3) \| 8.3 (6.7, 9.3) \| 8.0 (6.3, 9.3) \|  \| \| Engagement(Average) \|  \|  \| 0.022 \| \| Median (Q1, Q3) \| 8.0 (7.0, 9.0) \| 7.7 (6.7, 9.0) \|  \| \| Relationships(Average) \|  \|  \| 0.896 \| \| Median (Q1, Q3) \| 8.3 (7.0, 9.3) \| 8.3 (7.0, 9.3) \|  \| \| Meaning(Average) \|  \|  \| 0.817 \| \| Median (Q1, Q3) \| 8.3 (6.7, 9.3) \| 8.3 (6.7, 9.3) \|  \| \| Accomplishments(Average) \|  \|  \| 0.461 \| \| Median (Q1, Q3) \| 8.0 (6.3, 9.3) \| 8.0 (6.3, 9.0) \|  \| \| Externalizing Behavior Score \|  \|  \| 0.698 \| \| Median (Q1, Q3) \| 5.0 (3.0, 7.0) \| 5.0 (2.0, 7.0) \|  \| \| Internalizing Behavior Score \|  \|  \| 0.788 \| \| Median (Q1, Q3) \| 5.0 (3.0, 8.0) \| 5.0 (3.0, 7.0) \|  \| \| Department \|  \|  \| 0.053 \| \| Science \| 180 (56.4%) \| 101 (47.2%) \|  \| \| Arts \| 103 (32.3%) \| 91 (42.5%) \|  \| \| Technology \| 36 (11.3%) \| 22 (10.3%) \|  \| \| Nationality \|  \|  \| 0.434 \| \| Qatari \| 127 (39.8%) \| 78 (36.4%) \|  \| \| Non-Qatari \| 192 (60.2%) \| 136 (63.6%) \|  \| \| Do you consider the economic/financial condition of your family? \|  \|  \| 0.025 \| \| Below Average \| 52 (16.3%) \| 53 (24.8%) \|  \| \| Average \| 210 (65.8%) \| 118 (55.1%) \|  \| \| Above Average \| 57 (17.9%) \| 43 (20.1%) \|  \| \| What is your academic level? \|  \|  \| 0.053 \| \| Below Average \| 12 (3.8%) \| 9 (4.2%) \|  \| \| Average \| 44 (13.8%) \| 37 (17.3%) \|  \| \| Good \| 119 (37.3%) \| 97 (45.3%) \|  \| \| Excellent \| 144 (45.1%) \| 71 (33.2%) \|  \| \| Are you absent from school? \|  \|  \| 0.011 \| \| Often \| 3 (0.9%) \| 9 (4.2%) \|  \| \| Sometimes \| 43 (13.5%) \| 43 (20.1%) \|  \| \| Rarely \| 118 (37.0%) \| 67 (31.3%) \|  \| \| Never \| 155 (48.6%) \| 95 (44.4%) \|  \| |

## Table S3: Patient demographic, PERMA, and SDQ characteristics stratified by SES

| \|  \| Below Average \| Average \| Above Average \|  \| \| --- \| --- \| --- \| --- \| --- \| \|  \| (N = 105) \| (N = 328) \| (N = 100) \| p-value \| |
| --- | --- | --- | --- | --- | --- | --- | --- | --- | --- | --- |
| \| PERMA (Average) \|  \|  \|  \| <0.001 \| \| --- \| --- \| --- \| --- \| --- \| \| Median (Q1, Q3) \| 111.5 (91.5, 124.7) \| 110.6 (94.5, 125.1) \| 121.6 (109.4, 130.7) \|  \| \| Emotion(Average) \|  \|  \|  \| <0.001 \| \| Median (Q1, Q3) \| 8.0 (6.0, 9.0) \| 8.0 (6.3, 9.0) \| 9.0 (8.0, 10.0) \|  \| \| Engagement(Average) \|  \|  \|  \| 0.005 \| \| Median (Q1, Q3) \| 7.7 (6.7, 9.0) \| 8.0 (6.7, 9.0) \| 8.3 (7.5, 9.2) \|  \| \| Relationships(Average) \|  \|  \|  \| 0.001 \| \| Median (Q1, Q3) \| 8.3 (6.7, 9.3) \| 8.0 (6.7, 9.3) \| 9.0 (8.0, 9.7) \|  \| \| Meaning(Average) \|  \|  \|  \| <0.001 \| \| Median (Q1, Q3) \| 8.0 (6.3, 9.3) \| 8.0 (6.7, 9.3) \| 9.0 (7.7, 10.0) \|  \| \| Accomplishments(Average) \|  \|  \|  \| 0.003 \| \| Median (Q1, Q3) \| 8.0 (6.0, 9.0) \| 8.0 (6.2, 9.0) \| 8.7 (6.7, 9.7) \|  \| \| Externalizing Behavior Score \|  \|  \|  \| 0.306 \| \|  \|  \|  \|  \|  \| \| Median (Q1, Q3) \| 4.0 (2.0, 7.0) \| 5.0 (3.0, 7.0) \| 4.0 (2.0, 6.5) \|  \| \| Internalizing Behavior Score \|  \|  \|  \| 0.006 \| \| Median (Q1, Q3) \| 5.0 (3.0, 8.0) \| 5.0 (3.0, 8.0) \| 4.0 (3.0, 6.0) \|  \| \| Department \|  \|  \|  \| 0.025 \| \| Science \| 52 (49.5%) \| 186 (56.7%) \| 43 (43.0%) \|  \| \| Arts \| 45 (42.9%) \| 110 (33.5%) \| 39 (39.0%) \|  \| \| Technology \| 8 (7.6%) \| 32 (9.8%) \| 18 (18.0%) \|  \| \| Nationality \|  \|  \|  \| <0.001 \| \| Qatari \| 24 (22.9%) \| 116 (35.4%) \| 65 (65.0%) \|  \| \| Non-Qatari \| 81 (77.1%) \| 212 (64.6%) \| 35 (35.0%) \|  \| \|  \|  \|  \|  \|  \| \|  \|  \|  \|  \|  \| \|  \|  \|  \|  \|  \| \|  \|  \|  \|  \|  \| \| What is your academic performance level? \|  \|  \|  \| 0.541 \| \| Below Average \| 6 (5.7%) \| 12 (3.7%) \| 3 (3.0%) \|  \| \| Average \| 14 (13.3%) \| 55 (16.8%) \| 12 (12.0%) \|  \| \| Good \| 48 (45.7%) \| 124 (37.8%) \| 44 (44.0%) \|  \| \| Excellent \| 37 (35.2%) \| 137 (41.8%) \| 41 (41.0%) \|  \| \| Are you absent from school? \|  \|  \|  \| 0.211 \| \| Often \| 3 (2.9%) \| 6 (1.8%) \| 3 (3.0%) \|  \| \| Sometimes \| 15 (14.3%) \| 51 (15.5%) \| 20 (20.0%) \|  \| \| Rarely \| 31 (29.5%) \| 112 (34.1%) \| 42 (42.0%) \|  \| \| Never \| 56 (53.3%) \| 159 (48.5%) \| 35 (35.0%) \|  \| |

## Table S4: Patient demographic, PERMA, and SDQ characteristics stratified by academic level

| \|  \| Below Average \| Average \| Good \| Excellent \|  \| \| --- \| --- \| --- \| --- \| --- \| --- \| \|  \| (N = 21) \| (N = 81) \| (N = 216) \| (N = 215) \| p-value \| |
| --- | --- | --- | --- | --- | --- | --- | --- | --- | --- | --- | --- | --- |
| \| PERMA (Average) \|  \|  \|  \|  \| <0.001 \| \| --- \| --- \| --- \| --- \| --- \| --- \| \| Median (Q1, Q3) \| 80.7 (46.3, 99.3) \| 108.5 (83.5, 119.4) \| 113.6 (96.4, 126.6) \| 116.7 (103.3, 129.7) \|  \| \| Emotion(Average) \|  \|  \|  \|  \| <0.001 \| \| Median (Q1, Q3) \| 6.0 (4.0, 7.0) \| 7.7 (5.3, 9.0) \| 8.3 (6.8, 9.3) \| 8.3 (7.0, 9.3) \|  \| \| Engagement(Average) \|  \|  \|  \|  \| <0.001 \| \| Median (Q1, Q3) \| 6.3 (4.3, 7.7) \| 8.0 (6.3, 9.0) \| 8.0 (6.7, 9.0) \| 8.3 (7.0, 9.3) \|  \| \| Relationships(Average) \|  \|  \|  \|  \| 0.005 \| \| Median (Q1, Q3) \| 6.3 (4.7, 8.7) \| 7.7 (6.0, 9.3) \| 8.3 (7.0, 9.3) \| 8.3 (7.3, 9.3) \|  \| \| Meaning(Average) \|  \|  \|  \|  \| <0.001 \| \| Median (Q1, Q3) \| 4.7 (2.3, 6.3) \| 7.3 (5.3, 9.0) \| 8.0 (6.7, 9.3) \| 8.7 (7.7, 9.7) \|  \| \| Accomplishments(Average) \|  \|  \|  \|  \| <0.001 \| \| Median (Q1, Q3) \| 5.0 (2.3, 6.7) \| 7.0 (5.0, 8.7) \| 8.0 (6.3, 9.0) \| 8.3 (7.3, 9.7) \|  \| \| Externalizing Behavior Score \|  \|  \|  \|  \| <0.001 \| \| Median (Q1, Q3) \| 8.0 (3.0, 11.0) \| 7.0 (5.0, 9.0) \| 4.5 (2.0, 6.0) \| 4.0 (2.0, 6.0) \|  \| \| Internalizing Behavior Score \|  \|  \|  \|  \| <0.001 \| \| Median (Q1, Q3) \| 9.0 (7.0, 14.0) \| 6.0 (4.0, 10.0) \| 5.0 (3.0, 7.0) \| 5.0 (3.0, 8.0) \|  \| \| Department \|  \|  \|  \|  \| <0.001 \| \| Science \| 6 (28.6%) \| 24 (29.6%) \| 94 (43.5%) \| 157 (73.0%) \|  \| \| Arts \| 13 (61.9%) \| 47 (58.0%) \| 98 (45.4%) \| 36 (16.7%) \|  \| \| Technology \| 2 (9.5%) \| 10 (12.3%) \| 24 (11.1%) \| 22 (10.2%) \|  \| \| Nationality \|  \|  \|  \|  \| <0.001 \| \| Qatari \| 15 (71.4%) \| 49 (60.5%) \| 91 (42.1%) \| 50 (23.3%) \|  \| \| Non-Qatari \| 6 (28.6%) \| 32 (39.5%) \| 125 (57.9%) \| 165 (76.7%) \|  \| \| Do you consider the economic/financial condition of your family? \|  \|  \|  \|  \| 0.541 \| \| Below Average \| 6 (28.6%) \| 14 (17.3%) \| 48 (22.2%) \| 37 (17.2%) \|  \| \| Average \| 12 (57.1%) \| 55 (67.9%) \| 124 (57.4%) \| 137 (63.7%) \|  \| \| Above Average \| 3 (14.3%) \| 12 (14.8%) \| 44 (20.4%) \| 41 (19.1%) \|  \| \| What is your academic level? \|  \|  \|  \|  \| <0.001 \| \| Below Average \| 21 (100.0%) \| 0 (0.0%) \| 0 (0.0%) \| 0 (0.0%) \|  \| \| Average \| 0 (0.0%) \| 81 (100.0%) \| 0 (0.0%) \| 0 (0.0%) \|  \| \| Good \| 0 (0.0%) \| 0 (0.0%) \| 216 (100.0%) \| 0 (0.0%) \|  \| \| Excellent \| 0 (0.0%) \| 0 (0.0%) \| 0 (0.0%) \| 215 (100.0%) \|  \| \| Are you absent from school? \|  \|  \|  \|  \| <0.001 \| \| Often \| 4 (19.0%) \| 0 (0.0%) \| 7 (3.2%) \| 1 (0.5%) \|  \| \| Sometimes \| 3 (14.3%) \| 25 (30.9%) \| 43 (19.9%) \| 15 (7.0%) \|  \| \| Rarely \| 8 (38.1%) \| 23 (28.4%) \| 87 (40.3%) \| 67 (31.2%) \|  \| \| Never \| 6 (28.6%) \| 33 (40.7%) \| 79 (36.6%) \| 132 (61.4%) \|  \| |
